# Bioluminescence imaging reveals inhibition of tumor cell proliferation by Alzheimer's amyloid β protein

**DOI:** 10.1186/1475-2867-9-15

**Published:** 2009-06-01

**Authors:** Hong Zhao, Jinmin Zhu, Kemi Cui, Xiaoyin Xu, Megan O'Brien, Kelvin K Wong, Santosh Kesari, Weiming Xia, Stephen TC Wong

**Affiliations:** 1 Center for Biotechnology and Informatics, The Methodist Hospital Research Institute and Department of Radiology, The Methodist Hospital, Weill Cornell Medical College, Houston, Texas 77030, USA; 2 Department of Radiology, Brigham and Women's Hospital, Harvard Medical School, Boston, Massachusetts 02115, USA; 3 Center for Neurologic Disease, Department of Neurology, Brigham and Women's Hospital, Harvard Medical School, Boston, Massachusetts 02115, USA; 4 Department of Medical Oncology, Dana Farber Cancer Institute, Harvard Medical School, Boston, Massachusetts 02115, USA; 5 Department of Neurology, Brigham and Women's Hospital, Harvard Medical School, Boston, Massachusetts 02115, USA

## Abstract

**Background:**

Cancer and Alzheimer's disease (AD) are two seemingly distinct diseases and rarely occur simultaneously in patients. To explore molecular determinants differentiating pathogenic routes towards AD or cancer, we investigate the role of amyloid β protein (Aβ) on multiple tumor cell lines that are stably expressing luciferase (human glioblastoma U87; human breast adenocarcinoma MDA-MB231; and mouse melanoma B16F).

**Results:**

Quantification of the photons emitted from the MDA-MB231 or B16F cells revealed a significant inhibition of cell proliferation by the conditioning media (CM) derived from amyloid precursor protein (APP) over-expressing cells. The inhibition of U87 cells was observed only after the media was conditioned for longer than 2 days with APP over-expressing cells.

**Conclusion:**

Our results suggest that Aβ plays an inhibitory role in tumor cell proliferation; this effect could depend on the type of tumor cells and amount of Aβ.

## Introduction

Cancer and Alzheimer's disease (AD) are the leading causes of death in the elderly. AD is characterized by progressive neuronal loss and associated deposition of amyloid-β proteins (Aβ) and neurofibril tangles. Glioblastoma multiforme (GBM) is the most malignant brain tumor in adults, and is one of the most devastating forms of cancer arising from dramatic proliferation and migration of tumor cells. Breast cancer is the second most fatal cancer in women, and skin cancer is the most common form of cancer. Like most diseases, the risk of getting AD or cancer increases with age. AD affects nearly 50% for those aged 85 years or older [1]. GBM reaches its highest levels between 65 and 85 years of age [2]. About 7% of population aged 60–79 has breast cancer, and this doubles when they reach 90 years old. In addition, it is estimated that about 50% people who live to age 65 or older have skin cancer at least once. A large population of cancer patients may not survive long enough to develop AD, especially in the cases of GBM. Among survivors of breast cancer or skin cancer, it seems that they rarely develop AD simultaneously. In addition, cancer occurs less frequently in AD patients than in the general population [3-5]. While epidemiologic and genetic studies have so far failed to prove that AD and cancer are likely to be mutually exclusive, decreased cancer frequency in AD patients would suggest certain cellular mechanisms may exist to prevent the co-occurrence of cancer and AD.

Amyloid β protein (Aβ) is generated from amyloid precursor protein (APP). APP is processed by two alternative cellular pathways. α-Secretase is involved in non- amyloidogenic processing and cleaves APP within the Aβ domain, whereby release of Aβ is prevented, and soluble APPsα is secreted. Amyloidogenic processing is driven by β-secretase, which cleaves APP at the N-terminal site of the Aβ domain. Both α- and β-secretase cleavages produce C-terminal stubs (termed C83 and C99, respectively) that remain inserted in the membrane and are either degraded or further processed by γ-secretase to produce the p3 fragment or the Aβ peptide, respectively [6,7].

Mounting evidence favors the amyloid cascade hypothesis that causally links AD pathological process and neuronal cell death to the aggregation and deposition of Aβ [8]. It has been reported that Aβ exhibits neurotoxic effect via multi-processes, and some are thought to involve generation of reactive oxygen species, alteration of intracellular calcium homeostasis and mitochondrial function, and activation of caspases [9]. Small, soluble oligomers of Aβ have been linked to neuronal toxicity and synaptic failure, as increased levels of oligomeric Aβ species in the media of cultured cells impaired hippocampal long-term potentiation [10].

APP processing is ubiquitous, and Aβ is produced by almost all types of cells. Aβ generation may be a defensive consequence of an underlying disease mechanism [9]. Ischemia, hypoglycemia and traumatic brain injury, conditions that have been shown to put neurons under metabolic stress, all up regulate APP expression in animal models and cultured cells [11,12]. They also re-route the metabolism of APP from the non-amyloidogenic to the amyloidogenic pathway. Inhibition of mitochondrial energy metabolism alters the processing of APP to generate amyloidogenic derivatives [13,14], and oxidative stress has been shown to increase the generation of Aβ [15-17]. The increased expression of APP and generation of Aβ under conditions of energetic stress may therefore be a response to the oxidative challenge observed in the brain in AD and following injury. In addition, it has been reported that APP may be related to the malignant progression of human astrocytic tumors [18], and it has been shown that intra-tumoral injection of Aβ potently inhibits the angiogenesis of human glioblastoma and thus inhibits the growth of the tumor [19].

To investigate whether naturally secreted Aβ has any effects on tumor cell proliferation, we have taken advantage of the APP over-expressing cells that generate high levels of Aβ. Conditioning media (CM) from APP-expressing cells contain all kinds of component of the APP derivatives, including Aβ monomers, oligomers, and soluble APPsα and APPsβ. For Aβ species, studies with any individual form of Aβ are difficult, as the different forms are always in equilibrium [20].

Here we adopted the bioluminescence imaging (BLI) for cell proliferation assay. BLI is a new technology in comparison to more popular cell proliferation assays, such as MTT assay [21]. It is highly sensitive for non-invasive examination of ongoing biological processes in small animals [22]. BLI is based on the detection of visible light produced during enzyme mediated oxidation of a substrate when the enzyme is expressed as a reporter. For cell-based assay, the imaging system can accommodate up to six 96-well plates in one imaging session, so it provides an ideal high-throughput screening tool to study cell proliferation provided that the cells express the reporter enzyme luciferase.

In this study we examined the effects of CM containing Aβ from APP over-expressing cells on three tumor cell lines, i.e., human GBM, breast cancer and mouse melanoma cells. Dynamic BLI cell proliferation assay was applied to measure the proliferation of these cells that stably express firefly luciferase. Aβ containing CM were obtained from Chinese Hamster Ovarian (CHO) and human neuroblastoma SH-SY5Y cells expressing wild type APP, and human embryonic kidney 293 (HEK293) cells expressing familial AD-linked Swedish mutant APP. We found that tumor cell proliferation was inhibited after the Aβ enriched CM was applied. This rapid assay makes it an attractive technique for high throughput screening for anti-proliferation compounds in the future.

## Methods

### Cell lines

Three cancer cell lines derived from human glioblastoma (U87-L), breast cancer (MDA-MB231-L) and mouse melanoma (B16F-L) that stably express firefly luciferase were daily monitored by BLI. The U87-L cells were generated as described previously [23]. The coding sequences for luciferase and neomycin phosphotransferase were fused and introduced into a pMMP retrovirus. The B16F-L cells were derived from murine non brain-metastatic melanoma tumor cells transduced with a lentiviral construct containing the firefly luciferase gene and the GFP gene [24]. The MDA-MB231-L cells were purchased from Xenogen (Xenogen Corporation, Alameda, CA). It is derived from MDA-MB231 human adenocarcinoma cells by stable transfection of the luciferase gene under the SV40 promoter.

CHO cells expressing wild type APP (7W), SH-SY5Y cells expressing wild type APP (SKAPP), HEK293 cells expressing human soluble APPsα (hAPPs) and HEK293 expressing familial AD-linked Swedish mutant APP (SW293) have been characterized previously [25-28]. The CHO, SH-SY5Y and HEK293 cells expressing endogenous APP were used as controls.

All cells were grown in DMEM (InVitrogen) medium supplemented with 10% fetal bovine serum (Sigma), 2 mmol/L L-glutamine (InVitrogen), 100 units/ml penicillin/streptomycin (InVitrogen), and in a humidified atmosphere at 37°C in 5% CO_2_.

### Media replacement

The U87-L, MDA-MB231-L, B16F-L cells (500 cells/well) and the 7W, CHO, SKAPP, SH-SY5Y, SW293, HEK293 and hAPPs, HEK293 cells (1000 cells/well) were seeded in separate 96 well plates. The CM from 7W, CHO, SKAPP, SH-SY5Y, SW293, HEK293 and hAPPs, HEK293 cells were harvested at 36 hr, 60 hr and 84 hr after being planted. Media from the tumor cells was removed and replaced with fresh CM. Tumor cells with the CM were monitored for 24 hr to 72 hr by BLI.

### Aβ assay by ELISA

The Aβ levels in CM were measured before media replacement. Aβ sandwich ELISAs were performed as described [29]. Aβ_40 _and Aβ_42 _levels were measured using a standard sandwich ELISA assay where 2G3 (to Aβ residues 33–40) and 21F12 (to Aβ residues 33–42) antibodies captured Aβ_40 _and Aβ_42_, respectively, and biotinylated 266 antibody was used for detection.

### Quantification of the number of viable cells by bioluminescence assay

To eliminate the difference in the level of luciferase expression in each cell line, calibration of photon flux with respect to actual cell count was performed. MDA-MB231-L, B16F-L, and U87-L cells in 100 μL media were seeded into a 96 well plate by serial dilutions from 51,200 cells to 50 cells per well, each repeated with 3 independent experiments. The plate was imaged using the IVIS 100 system (2 min, 8 bin, and level B/FOV 15 × 15 cm) (Xenogen Corporation, Alameda, CA) at 10 minutes after addition of luciferin at the final concentration of 150 μg/mL. Media without luciferin were served as negative control. The photon flux was linearly regressed on the actual cell count to obtain a calibration curve for each cell line, as previously reported [30].

The baseline of the tumor cell proliferation was obtained by BLI assay 12 hrs after cells were planted. Lights emitted from tumor cells were acquired until 72 hr post media replacement with 24 hr interval. An average of 3 kinetic bioluminescent acquisitions was obtained within 10 minutes for each acquisition time point. Regions of interest (ROI) were drawn over wells automatically and quantified by Living Image Software version 2.20. Data was analyzed based on total photon flux emission (photons/s) subtracted by the background photon flux of each well for the dark current measurement.

### Statistical analysis

SPSS10.0 software was used for the statistical analysis. Data was obtained from three independent experiments with duplicate determinations and expressed as means ± SEM (standard error of the means). Statistical analysis was performed by one-way ANOVA followed by Fisher's post-hoc test procedure for significance (*P *< 0.05).

## Results

### Stable expression of luciferase in U87-L, MB-231-L and B16F-L cells

**Figure 1:**
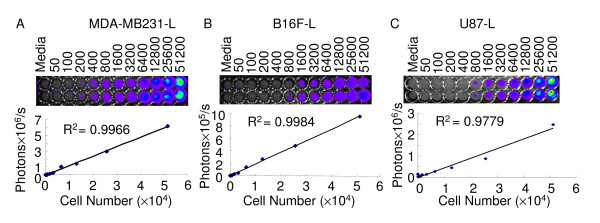
**Quantification of MDA-MB231-L, B16F-L and U87-L cell proliferation by bioluminescent imaging (BLI) assay**. MDA-MB231-L (A), B16F-L (B) and U87-L (C) cells in 100 μL media were seeded into a 96 well plate by serial dilutions from 51,200 cells to 50 cells per well. The plate was imaged using the IVIS system (2 min, 8 bin, and level B/FOV 15 × 15 cm) at 10 minutes after the addition of luciferin to final concentration of 150 μg/mL. Medium without luciferin was served as a negative control. A linear correlation between cell number and bioluminescent signal was obtained.

To quantify the cell proliferation based on the detected photons from the living cells, we have determined the correlation between the number of U87-L, MDA-MB231-L and B16F-L cells and the expression levels of luciferase using BLI assay. Our BLI apparatus reaches a sensitivity of detecting about 20 MDA-MB231-L cells. Due to different expression levels of luciferase gene and morphological appearance of cell types, as low as 80 to 110 of U87-L and B16F-L cells could be detected by our BLI apparatus. When cells were seeded at higher density, there was no saturation of signals using the above imaging parameters. A positive linear correlation between the cell numbers and the BLI signals was obtained, and all three cell lines have demonstrated clear correlation, i.e., R^2 ^= 0.9779–0.9984 (Fig. 1). Therefore, it is reliable to quantify the number of viable cells and cell proliferation based on the photon emission detected by BLI.

### Conditioning media from APP over-expressing CHO cells reduced the proliferation of MB-231-L cells

To explore any molecular determinants that differentiate pathways leading to AD versus cancer, we explore the role of the key peptide found in the brains of AD patients, Aβ, which secretes from different types of cells and aggregates into extracellular neuritic plaques. Instead of using synthetic Aβ peptide, we used naturally secreted Aβ in the conditioning media (CM) from three types of cells (CHO, HEK293 and SH-SY5Y) to treat tumor cells and searched for any inhibitory effect on cell proliferation.

**Figure 2:**
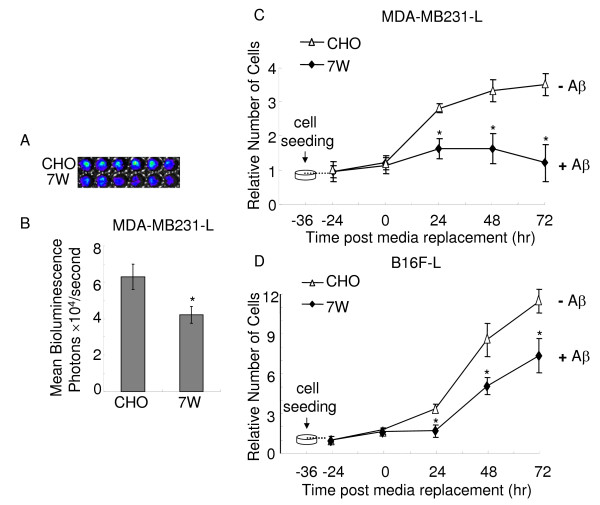
**Media from APP over-expressing CHO cells suppress MDA-MB231-L and B16F-L cell proliferation**. MDA-MB231-L and B16F-L cells (500 cells/well), APP over-expressing CHO cells (7W cells) and CHO cells (1000 cells/well) were seeded in 96 well plates. After 36 hr, media of MDA-MB231-L and B16F-L cells were replaced with the media from 7W or CHO cells. Proliferation of the MDA-MB231-L and B16F-L cells was measured by BLI assay at 24 hr post medium replacement. (A) A representative image from MDA-MB231-L cells cultured with conditioning media (CM) from CHO cells (CHO-CM) or from 7W cells (7W-CM) for 24 hr. 7W-CM and control CHO-CM were applied to 6 wells of adenocarcinoma MDA-MB231-L cells, respectively (top panel CHO-CM, lower panel 7W-CM). (B) Mean values from three independent experiments were obtained, and media from 7W cells showed inhibition of MDA-MB231-L proliferation, compared to media from CHO cells. The standard error of means (SEM) was illustrated (n = 12), and the asterisk indicates a statistically significant difference between two groups (*P *< 0.05, one-way ANOVA followed by Fisher's post-hoc test). (C) Persistent inhibitory effect of CM from 7W cells on MDA-MB231-L cell proliferation. The proliferation of the MDA-MB231-L cells was measured by BLI assay at 24, 48 and 72 hr post medium replacement. The photon counts at 24 hr before medium replacement (-24 hr) were used as the baseline for the calculation of relative cell numbers. All values indicate means ± SEM (n = 6), and significant difference between CM from 7W and control CHO cells is indicated by * *P *< 0.05. (D) Inhibitory effect of media from APP over-expressing CHO cells on melanoma (B16F-L) cell proliferation. B16F-L cells were quantified by BLI assay at 24, 48 and 72 hr after incubation with CM from 7W or CHO cells. All values represent means ± SEM (n = 6), and CM from 7W cells showed significant difference in it ability to inhibit B16F-L cell proliferation compared to CM from control CHO cells, as indicated by * *P *< 0.05.

We conditioned APP-over-expressing cells (7W cells) and its parental cell line CHO cells for 36 hrs, and harvested Aβ-rich 7W CM and control CHO CM, then applied them to 6 wells of adenocarcinoma MDA-MB231-L cells, respectively (Fig. 2A, top panel CHO CM, lower panel 7W CM). The effect of Aβ on tumor cell proliferation was measured by BLI (Fig. 2A). After incubation for 24 hr, conditioning media from 7W cells significantly delayed MDA-MB231-L cell proliferation compared to media from control CHO cells (P < 0.05) (Fig. 2B). When we continued to incubate tumor cells with CM from CHO or 7W cells for 48 hrs, the number of MDA-MB231-L cells incubated with CM from 7W cells was significantly lower than those incubated with control CHO CM (Fig. 2C). Extended incubation up to 72 hr further decelerated cell proliferation, resulting in about 50% reduction in the number of cells grown in 7W CM, compared to those in CHO CM (Fig. 2C). Together, these results suggested that CM from 7W cells reduced the proliferation of MDA-MB231-L cells.

### Inhibitory effect was independent of tumor cell types

To determine whether the inhibitory effect of the CM from 7W cells depends on the type of the tumor cell, we used a second cancer cell line: melanoma B16F-L. We conditioned 7W and CHO cells for 36 hr. After CM from 7W or CHO cells were applied to B16F-L cells and incubated for an extended period, we found a similar, but less dramatic effect on cell proliferation, as measured by BLI assay (Fig. 2D). At 24 hr post media replacement, the number of B16F-L cells grown in the CM from 7W cells was 50% ± 9% of those grown in CHO control media (Fig. 2D), while the number of MDA-MB231-L cells grown in the CM of 7W was 69% ± 5% of those grown in CHO CM (Fig. 2C). At 48 hr post medium replacement, numbers of B16F-L and MDA-MB231-L cells grown in 7W CM were 59% ± 6% and 59% ± 4% of those with CHO CM, respectively (Fig. 2C and 2D). The delay in cell proliferation was continued to 72 hr post medium replacement, when the number of cells in the presence of 7W CM was about 64% ± 7% and 47% ± 5% of those with CHO CM (Fig. 2C and 2D). Therefore, the CM from APP over-expressing 7W cells decelerated proliferation of both adenocarcinoma and melanoma cells, suggesting that the inhibition was independent of tumor cell types.

### Cell media conditioned for extended period had greater inhibitory effect on tumor cell proliferation

**Figure 3:**
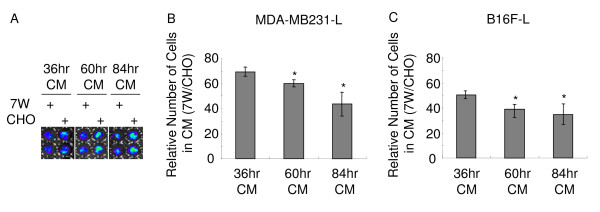
**Media conditioned for extended period with APP over-expressing CHO cells have greater inhibitory effect on tumor cell proliferation**. MDA-MB231-L cells, B16F-L cells (500 cells/well), 7W and CHO cells (1000 cells/well) were seeded in separate 96 well plates. 7W and control CHO cells were grown for 36 hr, 60 hr and 84 hr before the media were harvested for incubation with tumor cells. The number of tumor cells was measured by BLI assay at 24 hr post medium replacement. (A) A representative image of MDA-MB231-L cells was acquired at 24 hr after the media were replaced with media conditioned with CHO or 7W cells for 36, 60 or 80 hr. (B) The relative number of cells grown in CM from 7W vs. CHO was calculated by dividing MDA-MB231-L cells grown in 7W-CM by those in CHO-CM. The values represent means ± SEM (n = 6), and the difference is statistically significant compared to cells in 36 hr-CM, as indicated by * *P *< 0.05.

Since more Aβ is secreted into the CM with longer incubation time [31], we tested whether the media conditioned for a longer period would result in greater effect on tumor cell proliferation. In addition to 36 hr incubation period, we cultured 7W and CHO cells for 60 hr and 84 hr (conditioning times) then collected CM. Next, we applied the CM to MDA-MB231-L or B16F-L cells and incubated for 24 hr (incubation time) before acquiring images by BLI (Fig. 3A). For the MDA-MB231-L cells, the CM conditioned for 60 hr (60 hr-CM) showed stronger inhibitory effect than the 36 hr-CM (P < 0.05), and the 84 hr-CM showed the strongest inhibitory effect compared to the 36 hr- and 60 hr-CM (P < 0.05) (Fig. 3A and 3B). For the B16F-L cells, both 60 hr-CM and 84 hr-CM showed stronger inhibition compared to 36 hr-CM (P < 0.05), but no significant difference was observed between the 60 hr- and 84 hr-CM (Fig. 3C). Taken together, these results indicate that more Aβ accumulated in media conditioned for an extended period had greater inhibition on proliferation of tumor cells.

### Source of cells for preparing conditioning media was not cell type specific

**Figure 4:**
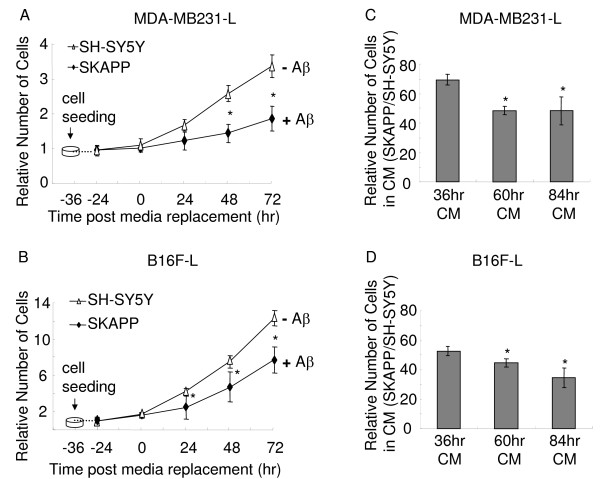
**APP over-expressing human neuroblastoma SH-SY5Y cells (SKAPP cells) had the similar inhibitory effects on the tumor cell proliferation**. MDA-MB231-L cells, B16F-L cells (500 cells/well) and SKAPP, SKNSH cells (1000 cells/well) were seeded in separate 96 well plates. The number of MDA-MB231-L cells (A) and B16F-L cells (B) was measured by BLI assay at 24, 48 and 72 hr after incubation of 36 hr-CM from SKAPP or SH-SY5Y cells. The media from SKAPP cells showed significant difference in their ability to inhibit tumor cell proliferation compared to the media from control SH-SY5Y cells, as indicated by * *P *< 0.05. (C and D) SKAPP and control SKNSH cells were grown for 36 hr, 60 hr and 84 hr for incubation with tumor cells. The number of tumor cells was measured by BLI assay after 24 hr of incubation. The inhibitory effect of media on MDA-MB231-L cells (C) and B16F-L cells (D) was stronger than those media from shorter period of conditioning. The difference is statistically significant, * *P *< 0.05. All values represent means ± SEM (n = 6).

In order to determine whether Aβ from other cell types had similar effects, media from the APP over-expressing human neuroblastoma SH-SY5Y cells (SKAPP cells) and its parental cell line SH-SY5Y were used for the incubation with tumor cells. After SKAPP and SH-SY5Y cells were grown for 36 hrs (conditioning time), CM was collected and applied on tumor cells for 24, 48 and 72 hr, followed by BLI acquisition. Apparently, there was a delay in cell proliferation in the presence of CM from SKAPP cells during the incubation period from 24 to 72 hrs. While the number of cells grown in the CM of SKAPP cells was statistically lower than those in the CM of SH-SY5Y cells, the difference in the number of MDA-MB231-L cells (Fig. 4A) was slightly larger than those of B16F-L cells (Fig. 4B). This is consistent with the previous observation with the CM from 7W and CHO cells (Fig. 2C and 2D).

Similarly, when we extended the conditioning time to 60 and 84 hrs, we found that CM from SH-SY5Y cells acted almost identically to the CM from CHO cells. The 60 hr- and 84 hr-CM from SKAPP cells showed stronger inhibition effect on cell proliferation of MDA-MB231-L and B16F-L cells than 36 hr-CM (P < 0.05), but no significant difference was observed between the media conditioned for 60 hr and 84 hr (Fig. 4C and 4D). This is highly similar to B16F-L cells grown in the CM from 7W cells (Fig. 3C).

### Soluble APP in conditioned media failed to inhibit cell proliferation

**Figure 5:**
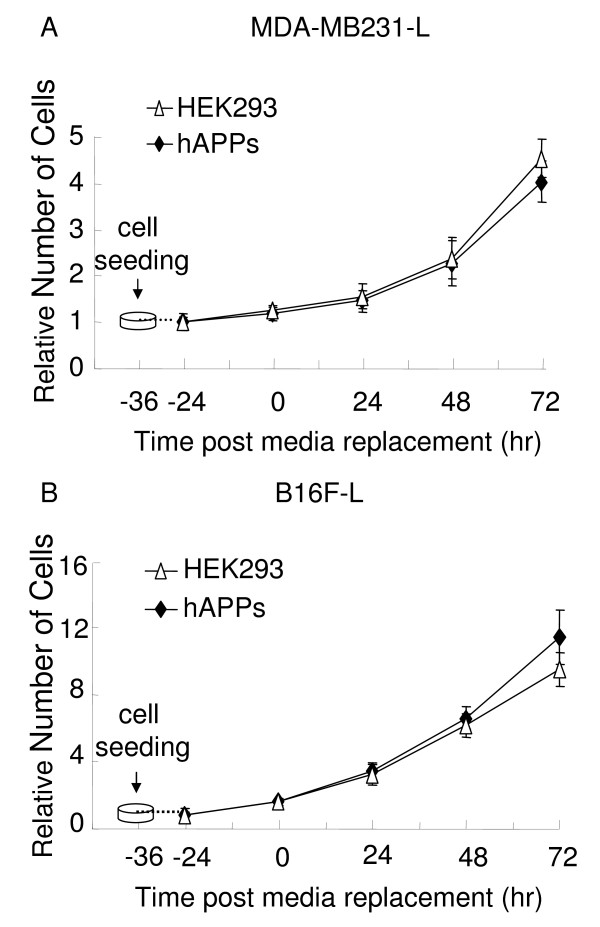
**Lack of inhibitory effect on cell proliferation by soluble APP in CM from hAPPs expressing cells**. MDA-MB231-L cells, B16F-L cells (500 cells/well), hAPPs and HEK293 cells (1000 cells/well) were seeded in separate 96 well plates. The number of MDA-MB231-L cells and B16F-L cells was measured by BLI assay after incubation of 36 hr-CM from hAPPs (A) or HEK293 cells (B). The media from hAPPs cells showed no significant difference in their ability to inhibit tumor cell proliferation compared to media from control HEK293 cells. All values represent means ± SEM (n = 6).

The major components secreted from APP over-expressing cells include Aβ as well as soluble APP (APPsα and APPsβ). The majority of soluble APP is APPsα, which derives from α-secretase cleavage of APP; only very low levels of APPsβ (derived from β-secretase cleavage of APP) exist in the CM. Therefore, we searched for any inhibitory effect on tumor cell proliferation by APPsα. Taking advantage of stable HEK293 cell line overexpressing soluble APPsα, hAPPs, we cultured hAPPs cells and its parental cell line HEK293. Because the α-secretase cleaves in the middle of Aβ region, thus precluding the generation of Aβ, only endogenous levels of Aβ are generated in hAPPs cells, and levels of Aβ in CM from hAPPs expressing cells were undetectable by our ELISA (data not shown). When we conditioned media from hAPPs and HEK293 cells for 36 hr and applied CM to MDA-MB231-L and B16F-L cells for 24, 48 and 72 hrs, no inhibitory effects were observed (Fig. 5A and 5B). Therefore, high levels of APPsα did not affect the cell proliferation.

### Inhibitory effect on tumor cell proliferation correlates with Aβ concentrations in conditioning media

**Figure 6:**
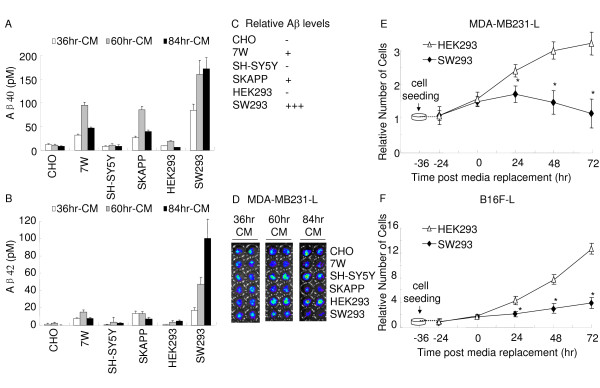
**Inhibitory effect of CM on tumor cell proliferation correlates with Aβ concentrations in the CM**. (A, B) 36 hr-, 60 hr- and 84 hr-CM from 7W, SKAPP, SW293 and CHO, SH-SY5Y, HEK293 cells were collected, and Aβ40 and Aβ42 concentrations were measured by ELISA. Much more Aβ40 (A) and Aβ42 (B) were produced by 7W, SKAPP and SW293 cells than their wild type parental cells (*P *< 0.05). The Aβ40 and Aβ42 concentrations in the SW293-CM were significantly higher than those in 7W-CM and SKAPP-CM(*P *< 0.05). All values represent means ± SEM (n = 12). (C) Relative levels of Aβ generated from each cell line were illustrated, based on the actual levels of Aβ (A, B). (D) Representative images from MDA-MB231-L cell cultured with 36 hr, 60 hr and 84 hr- CM from 7W/CHO, SKAPP/SH-SY5Y and SW293/HEK293 cells. (E, F) Persistent inhibitory effect of SW293-CM on MDA-MB231-L and B16F-L cells proliferation. The proliferation of the MDA-MB231-L and B16F-L cells were measured by BLI assay at 24, 48 and 72 hr post medium replacement. All values indicate means ± SEM (n = 6), and the difference between SW293 and control HEK293 cells is statistically significant, as indicated by *, *P *< 0.05.

To validate that Aβ in the CM delayed the proliferation of these tumor cells, we measured Aβ40 and Aβ42 concentrations in the CM from all cell lines. We found that both 7W and SKAPP secreted similar amount of Aβ into the CM. Therefore, we used a third cell line, a HEK293 cell line over-expressing familial AD-linked Swedish mutant APP (SW293). It has been reported that SW293 cells secrete very high levels of Aβ to the CM [28]. Clearly, within 36 hr, much more Aβ40 and Aβ42 were secreted into the CM from SW293 cells than those from 7W and SKAPP cells (Fig. 6A and 6B), and Aβ levels increased significantly upon prolonged conditioning for 60 hr (Fig. 6A and 6B). The amount of Aβ40 and Aβ42 in the CM from SW293 cells continued to increase and maintained at high levels when cells were conditioned to 84 hr, but the levels of Aβ in the CM from 7W and SKAPP cells started to decrease when conditioning was extended to 84 hrs (Fig. 6A and 6B), partially due to a potential degradation and/or aggregation of Aβ peptide, where our ELISA only measures soluble monomeric Aβ.

Similar to the findings that Aβ40 and Aβ42 concentrations in the CM from the SW293 cells were significantly higher than those from 7W and SKAPP cells (Fig. 6A, B and 6C), the inhibitory ability of CM from SW293 cells on MDA-MB231-L and B16F-L cells was stronger than those of 7W and SKAPP cell CM. Compared with HEK293 CM treated cells, the number of MDA-MB231-L and B16F-L cells were much less after 24 hr incubation in the 36 hr-CM from SW293 cells (68% ± 2% and 49% ± 4%, respectively). Longer periods of incubation up to 72 hr also showed reduced cell numbers to 41% ± 3% and 31% ± 3%, respectively (Fig. 6D, E and 6F).

### High levels of Aβ were necessary to inhibit the proliferation of human neuroblastoma cells (U87-L)

**Figure 7:**
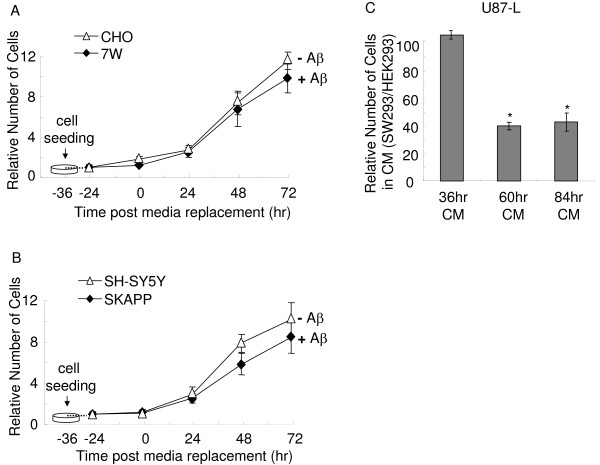
**Proliferation of human neuroblastoma cells (U87-L) is attenuated by conditioned media with high Aβ levels**. U87-L cells (500 cells/well) and 7W/CHO, SKAPP/SH-SY5Y, SW293/HEK293 cells (1000 cells/well) were seeded in separate 96 well plates. The U87-L cells were measured by BLI assay at 24, 48 and 72 hr after incubation of 36 hr-CM. No inhibitory effect was found by the 36 hr-CM from 7W cells (A) and SKAPP cells (B). (C) CM were harvested after SW293 and HEK293 cells were grown for 36 hr, 60 hr and 84 hr, The inhibitory effect of 60 hr and 84 hr SW293 CM on U87-L cells was stronger than the ability of 36 hr-CM. All values represent means ± SEM (n = 6), and the difference is statistically significant, * *P *< 0.05 compared with 36 hr-CM.

Apparently, both MDA-MB231-L and B16F-L were very sensitive to Aβ in the CM. Next, we examined a cell line that seemed to be less sensitive to Aβ in the CM, human glioblastoma, U87-L. When BLI was used to monitor the proliferation of U87-L upon the incubation of cells in the CM from 7W and SKAPP cells, no inhibitory effects were observed because these cells produced relatively low Aβ compared to SW293 cells (Fig. 7A and 7B). However, we found a significant delay in cell proliferation by the SW293 CM that was conditioned for 60 hr or 84 hr (Fig. 7C). We did not observe much inhibitory effect by the CM conditioned for 36 hr, consistent with the fact that the Aβ40 and Aβ42 levels of the 60 hr-CM and 84 hr-CM are two to five fold greater than that in the 36 hr-CM from SW293 cells (Fig. 6A and 6B). These results indicate that inhibitory ability of the CM correlated to the concentration of Aβ levels.

### Surface plots of tumor cell proliferation versus the concentration of Aβ40 and Aβ42 in the conditioning media

**Figure 8:**
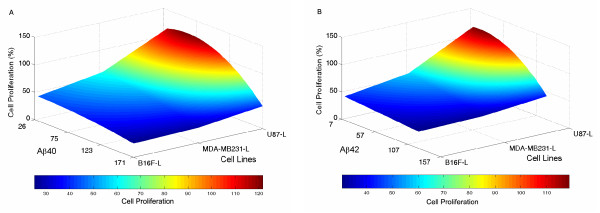
**Surface plots of tumor cell proliferation versus the concentration of Aβ40 and Aβ42 in the CM**. Second-order polynomial was used to approximate the relationship between the tumor cell proliferation and Aβ40 (A), Aβ42 (B) concentrations. Using the derived polynomial, the tumor cell proliferation from minimum Aβ40 and Aβ42 concentrations to maximum concentrations at a step size of 5 was calculated. The Matlab^® ^command "surf" was used to plot the proliferation of three tumor cell lines as a function of the Aβ40 and Aβ42 concentrations. The region of high cell proliferation (red zone) lies at low Aβ concentrations.

Finally, we plotted the cell proliferation of the three tumor cell lines as a function of the Aβ concentration (Fig. 8). We used a second-order polynomial to approximate the relationship between the cell proliferation of B16F-L and Aβ40 concentrations. We sorted the Aβ concentrations in the ascending order for all the three time points (36 hr, 60 hr, and 84 hr), followed by a second-order polynomial to fit the corresponding cell proliferation of B16F-L to minimize the least-squares error. Using the derived polynomial, we calculated the cell proliferation of the tumor cell from a minimum Aβ40 concentration of 26 pM to a maximum of 172 pM at a step size of 5 pM. The same procedure was used to perform curve-fitting for MDA-MB231-L and U87-L cell lines. Finally we used the Matlab^® ^command "surf" to plot the cell proliferation of the three tumor cell lines as a function of the Aβ40 concentration (Fig. 8A). Identical approach was used to plot the cell proliferation versus the Aβ42 concentration (Fig. 8B). While three tumor cell lines exhibited different cell proliferation at the same Aβ concentrations, these tumor cells tended to have reduced cell proliferation as the concentration of Aβ increased.

## Discussion

The inhibitory effects on tumor cell proliferation attribute to Aβ in the conditioning media of the cells over-expressing wild type or Swedish mutant APP. Aβ peptides are released as soluble products from APP via a series of metabolic cleavage steps. After 36 hr of growth, concentrations of Aβ in the CM of 7W, SKAPP and SW293 cells were much higher compared with their corresponding controls, CHO, SH-SY5Y, and HEK293. The inhibitory effects on the proliferation of breast and skin cancer cells were observed upon incubation with the Aβ enriched CM for 24 hrs. It has been shown that various species of Aβ possess different degrees of cellular toxicity. Soluble Aβ oligomers, including amyloid-derived diffusible ligands (ADDLs), are more neurotoxic than fibrils and insoluble larger aggregates [32,33]. A variety of Aβ species carry different physiochemical and pharmacological properties, and soluble monomers [34], low molecular weight oligomers and ADDLs [10,35], and protofibrils [20,36] are in equilibrium with insoluble, high molecular weight fibrils [20]. Aβ42 is known to be more amyloidogenic than Aβ40. Additional factors, such as pH, ions (Cu^2+^, Fe^2+^, Zn^2+^) also influence the aggregation process [37-40]. In this study, it is expected that there was Aβ heterogeneity in aggregation states in the final CM. Our ELISA only detects soluble monomeric Aβ and cannot quantify the aggregated Aβ species. Therefore, it is difficult to determine the exact species that played the dominant inhibitory effects on the tumor cell proliferation. However, it is well known that synthetic Aβ peptides added in growth media are notoriously heterogeneous due to variable preparation conditions, and the status of Aβ peptide would continuously change during the cancer cell treatment period as they would oligomerize and aggregate. Therefore, naturally synthesized, cultured cell derived Aβ peptides have been used in our experiments, and this approach has been widely used for Aβ toxicity studies in AD research [10,41,42].

The CM naturally secreted from APP overexpressing cells contained substantial amounts of monomeric Aβ and some oligomeric Aβ. In viewing of the Aβ concentrations in the CM of 7W, SKAPP and SW293 cells, which showed a marked increase paralleled with the growth period up to 60 hrs and no further increase up to 84 hrs (except for Aβ42 from SW293 cells), it is possible that the soluble Aβ might aggregate into insoluble compositions as more monomeric Aβ was secreted from the cells during the extended conditioning period. We found a stronger inhibition of the tumor cells proliferation by the 60 hr-CM, compared to the effect by the 36 hr-CM. The CM from SW293 cells had stronger inhibition of the MDA-MB231-L and B16F-L cells than the 60 hr-CM. Meantime, we noticed that the media conditioned with three APP over expressing cells for 36 hrs had a persistent inhibition on tumor cell proliferation. The inhibitory effects on MDA-MB231-L and B16F-L cells became more prominent when the tumor cells were incubated with CM for a longer period. These Aβ species caused an inhibition of tumor cell growth at different time points, whereas CM of untransfected cells had no effect, indicating that both monomeric and oligomeric Aβ are responsible for the specific effect.

Since it is still controversial whether Aβ induced toxicity is cell type dependent [43], we adopted three tumor cell lines from different sources, the human breast cancer cell MDA-MB231, GBM cell U87, and mouse melanoma cell B16F. The MDA-MB231 and B16F cells showed high sensitivities to Aβ in the CM. The cell proliferation of U87, however, was attenuated only by the media with high level of Aβ. Although Aβ circulates in peripheral organs through the whole body, it is equally important to examine any effect of Aβ containing CM on brain tumor cells due to the fact that Aβ accumulates extensively in brains of AD patients. Our results have demonstrated that naturally secreted Aβ from mammalian cells directly inhibit the proliferation of tumor cells. It is not clear whether any Aβ receptors at cell surface facilitate the process, as microglia cells have been reported to promote receptor dependent Aβ uptake [44]. Previous studies have shown that G-protein-coupled formyl peptide receptor like 1 in glial cells mediates the uptake of its agonist Aβ42, and this process could be activated by other ligands [45]. For example, CpG-containing oligodeoxynucleotide, a Toll-like receptor 9 ligand, increases the expression and activity of formyl peptide receptor that leads to enhanced endocytosis of Aβ [45]. It is likely that a portion of Aβ in the conditioned media was endocytosed by U87 for subsequent degradation, which reduced the effect of Aβ on cell proliferation.

Our studies have taken advantage of the cell-based BLI assay that allows us to rapidly measure cell proliferation with excellent sensitivity at a high throughput scale. Although it requires luciferase expressing cell lines to examine the property of cell colonies, it is a powerful approach to estimate cell growth with great flexibility, e.g., it can be used for primary screening of the cytostatic activity of compounds.

Although our results would suggest that AD patients might be at lower risk for cancer occurrence, there is no strategy to overcome the risk of having AD by increasing Aβ levels in a hope to decrease the chance of cancer formation. In addition, these findings are obtained from cultured cells *in vitro*, while the micro-environment for tumor growth is more complicated *in vivo*. Therefore, simply modulating Aβ levels does not seem to be a solution for inhibiting tumor growth.

Nevertheless, understanding the association of Aβ with tumorigenesis is critical for the elucidation of molecular pathways related to cell proliferation. The key enzyme for the generation of Aβ, the γ-secretase, is the target not only for Alzheimer's disease but also for human T cell acute lymphoblastic leukemia [46]. While chemotherapy increases the activity and expression of the γ-secretase complex, the γ-secretase inhibitor renders colon cancer cells more sensitive to chemotherapy [47]. Importantly, the γ-secretase cleavage of APP generates two products, Aβ and the amyloid intracellular domain (AICD), the latter to be found as a regulator of EGF receptor at the transcription level [48]. EGF receptor is up-regulated in many types of tumors, therefore, both AICD and EGF receptor are explored at targets for cancer therapy. Furthermore, enhanced γ-secretase cleavage of APP leads to increased generation of APP C-terminal fragments, Aβ and AICD. It is conceivable that alteration of downstream gene expression caused by increased APP C-terminal fragments and AICD may lead to changes in proteins secreted to the conditioned media. Whether those novel factors inhibit cell proliferation awaits further exploration.

## Conclusion

This study provides evidence that naturally secreted Aβ inhibit the proliferation of cultured GBM, breast cancer, and skin cancer cells. Understanding the mechanism underlying the effects of Aβ on tumor cell proliferation would provide new pathways downstream of Aβ that may bind to putative tumor cell surface receptors. Identification of these pathways will reveal novel targets for therapeutic intervention of tumor cell growth.

## Abbreviations

AD: Alzheimer disease; Aβ: amyloid-β protein; APP: amyloid precursor protein; GBM: glioblastoma multiforme; AICD: APP intracellular domain; BLI: bioluminescence imaging; CHO: Chinese hamster ovary; HEK293: human embryonic kidney 293 cells; CM: conditioning media; ROI: region of interest; ADDLs: amyloid-derived diffusible ligands.

## Competing interests

The authors declare that they have no competing interests.

## Authors' contributions

HZ carried out cell culture experiments, HZ, JZ, XX, KC, and KW carried out BLI analyses, MO carried out Aβ measurements, HZ, SK, WX and SW participated in the design of the study, HZ, WX and SW conceived of the study and draft the manuscript. All authors read and approved the final manuscript.
